# Karyotype and reproduction mode of the rodent parasite *Strongyloides venezuelensis*

**DOI:** 10.1017/S0031182014001036

**Published:** 2014-08-04

**Authors:** AKINA HINO, TERUHISA TANAKA, MAHO TAKAISHI, YUMIKO FUJII, JUAN E. PALOMARES-RIUS, KOICHI HASEGAWA, HARUHIKO MARUYAMA, TAISEI KIKUCHI

**Affiliations:** 1 Division of Parasitology, Faculty of Medicine, University of Miyazaki, Miyazaki, 889-1692Japan; 2 Department of Infections, Respiratory and Digestive Medicine, Faculty of Medicine, University of the Ryukyus, Okinawa, 903-0213Japan; 3 Department of Environmental Biology, College of Bioscience and Biotechnology, Chubu University, Kasugai, Aichi, 487-8501Japan

**Keywords:** parthenogenesis, parasitic nematodes, karyotype

## Abstract

*Strongyloides venezuelensis* is a parasitic nematode that infects rodents. Although *Strongyloides* species described to date are known to exhibit parthenogenetic reproduction in the parasitic stage of their life cycle and sexual reproduction in the free-living stage, we did not observe any free-living males in *S. venezuelensis* in our strain, suggesting that the nematode is likely to depend on parthenogenetic reproduction. We confirmed by cytological analysis that *S. venezuelensis* produces eggs by parthenogenesis during the parasitic stage of its life cycle. Phylogenetic analysis using nearly the full length of 18S and D3 region of 28S ribosomal RNA gene suggested that *S. venezuelensis* is distantly related to another rodent parasite, namely *Strongyloides ratti*, but more closely related to a ruminant parasite, *Strongyloides papillosus*. Karyotype analysis revealed *S. venezuelensis* reproduces with mitotic parthenogenesis, and has the same number of chromosomes as *S. papillosus* (2*n* = 4), but differs from *S. ratti* (2*n* = 6) in this regard. These results, taken together, suggest that *S. venezuelensis* evolved its parasitism for rodents independently from *S. ratti* and, therefore, is likely to have a different reproductive strategy.

## INTRODUCTION

**Fig. 1.**
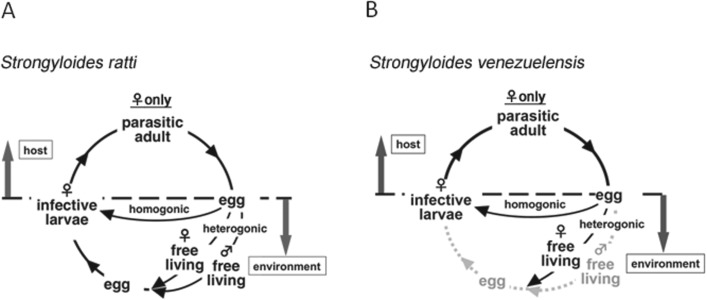
(A) Life cycle of *Strongyloides ratti*. Parasitic females produce eggs by parthenogenesis in the host's small intestine and the eggs are excreted into the environment in the faeces. Eggs develop into infective larvae by two alternative routes. In the homogonic route, eggs develop directly into infective larva. In the heterogonic route, eggs develop into free-living forms, reproducing sexually, after which the resultant eggs develop into infective larva. (B) Life cycle of *Strongyloides venezuelensis. S. venezuelensis* may lack the heterogonic developmental route in its life cycle.

The genus *Strongyloides* comprises over 50 species of nematodes that parasitize mammals, amphibians, reptiles and birds (Viney and Lok, 2007). *Strongyloides* nematodes have complex but interesting life cycles. Infection by *Strongyloides* begins when the infective third stage larvae (iL3) attach to and penetrate the host skin. Once inside the host, they moult twice into parasitic adults and settle in the small intestine of the host. Then, the parasitic adults, all female, produce eggs by parthenogenesis (Fig. 1A). Once the eggs or hatched larvae are excreted from the host, they develop via the homogonic route into iL3 forms or the heterogonic route into free-living stages that reproduce sexually outside the host (Fig. 1A); sexual reproduction in the free-living generation was shown for two species, *Strongyloides ratti* (Viney, 1996) and *Strongyloides papillosus* (Eberhardt *et al.* 2007). The progeny of free-living adults are entirely female and develop into iL3 forms in most *Strongyloides* species, including *S. ratti* and *Strongyloides stercoralis*, with a few exceptions (Yamada *et al.* 1991; Streit, 2008).

Parthenogenetic reproduction by parasitic females was found to be mitotic in cytological studies for *S. papillosus, Stronglyoides ransomi* (Triantaphyllou and Moncol, 1977) and for *S. ratti* (Chitwood and Graham, 1940), and using molecular markers for *S. ratti* (Viney, 1994) and *S. papillosus* (Nemetschke *et al.* 2010). In these species, the progeny from parasitic females can develop into one of three distinct morphologies: free-living females, free-living males (both via the heterogonic route) or iL3s (homogonic route). Although the ratio of these morphologies is influenced by environmental factors such as host immune response, temperature and pH (Arizono, 1976; Moncol and Triantaphyllou, 1978; Nwaorgu, 1983; Viney, 1996; Harvey *et al.* 2000; Minato *et al.* 2008; Sakamoto and Uga, 2013), the detailed mechanisms which determine their route of development remain unclear. Moreover, *S. stercoralis* and *S. ratti* harbour three pairs of chromosomes, and one of them is a sex (X) chromosome. Females of these species have an XX and free-living males have an XO karyotype (Streit, 2008). Genetic material homologous to two chromosomes in *S. ratti*, namely I and X, appears combined into one chromosome in *S. papillosus*. Additionally, an XX/XO karyotype appears to be functionally restored in males by a chromatin diminution event (Nemetschke *et al.* 2010).

*Strongyloides venezuelensis* parasitizes rodent species including rats, mice and Mongolian gerbil *Meriones unguiculatus* and is distributed worldwide (Brumpt, 1934; Wertheim and Lengy, 1964; Little, 1966; Hasegawa *et al.* 1988). *Strongyloides venezuelensis*, alongside *S. ratti*, is one of the most widely used laboratory models to study *Strongyloides* infection and mucosal immunity (Sato and Toma, 1990; El-Malky *et al.* 2013). Although both *S. ratti* and *S. venezuelensis* have rodents as hosts, the individual mechanisms by which they establish parasitism are thought to differ from each other. Phylogenetic analysis with ribosomal RNA gene suggested that *S. venezuelensis* is not as closely related to *S. ratti* as to *S. papillosus* and the primate parasite *Strongyloides fuelleborni* (Dorris *et al.* 2002). The migration patterns of the two rodent parasites in the host (Takamure, 1995), as well as mechanisms of host immunomodulation for successful parasitism, differ from each other as well (Wilkes *et al.* 2007; Matsumoto *et al.* 2013). More interestingly, observation of free-living adults is less common in *S. venezuelensis* as compared with *S. ratti* (Hasegawa *et al.* 1988; Harvey *et al.* 2000), suggesting that reproduction strategies used by the two species may also be different. Thus, the parasitic abilities of the two species may have evolved independently from each other, and a comparison between the two may provide invaluable insights into understanding the parasitic mechanism and its evolution in *Strongyloides* nematodes.

Though *S. venezuelensis* is a widely used laboratory model, some aspects of its basic biology remain unclear. In this report, we re-examined the phylogenetic position of *S. venezuelensis* within the genus *Strongyloides*, using 18S rRNA and the D3 expansion segment of 28S rRNA genes. We also examined the developmental routes of progeny from the parasitic females, chromosome behaviour in the germ cells and early embryos in *S. venezuelensis*. Our analyses suggest that acquisition of rodent parasitism occurred independently in *S. venezuelensis* and *S. ratti*.

## MATERIALS AND METHODS

### Culturing and handling nematodes

*Strongyloides venezuelensis* HH1 and *S. ratti* TDI isolates were used in this study. They were isolated from Okinawa, Japan (Hasegawa *et al.* 1988) and Tokyo, Japan, respectively, and maintained in the Parasitology laboratory of the University of Miyazaki, using male Wistar rats. Infectious aliquots were prepared by faecal culture using filter paper at 27 °C for 2 days and 5 days for *S. venezuelensis* and *S. ratti*, respectively (Sato and Toma, 1990). The nematodes were washed three times in distilled water, and administered by subcutaneous injection. *Strongyloides papillosus* iL3 was supplied from Dr Ayako Yoshida of University of Miyazaki. *Parastrongyloides trichosuri* DNA (strain KNP from Warwick Grant's lab) was supplied by Berriman lab from Wellcome Trust Sanger Institute, UK. *Strongyloides stercoralis* was collected in Yangon, Myanmar.

### PCR conditions and DNA sequencing

Nearly full-length 18S ribosomal DNA (rDNA) was amplified with primers 988F–1912R and 1813F–2646R from a lysate of a single nematode as described previously (Holterman *et al.* 2006). These primers amplify 1652 bp out of 1754 bp of full-length 18S rDNA of *C. elegans* (NR_000053·1). The D3 region of 28S rDNA was amplified using primers D3A-D3B (Nunn *et al.* 1996). PCR amplifications were carried out in 30 *μ*L reaction mixtures containing 15 *μ*L GoTaq Green Master Mix (Promega), 0·5 *μ*m of each primer, and 1 *μ*L of appropriately diluted nematode lysate under thermal-cycling conditions of 94 °C for 1 min, followed by 30 cycles of 94 °C for 30 s, 53 °C for 30 s and 72 °C for 1 min. PCR products were purified before sequencing using a MinElute 96 UF PCR purification plate (QIAGEN). DNA sequencing was performed using the BigDye Terminator 3.1 kit and ABI PRISM 3700 or 3130 Genetic Analyzer (Applied Biosystems).

### Phylogenetic analyses

Nearly full-length 18S rRNA gene and D3 expansion segments of 28S rRNA gene of *Strongyloides* species were used for phylogenetic reconstruction. *Parastrongyloides trichosuri* was used as an outgroup taxon. The newly obtained and published sequences for each gene were aligned using MAFFT (Katoh *et al.* 2002) with iterative refinement method (FFT-NS-i). Both datasets were concatenated using Concatenator v.1.1.0. (Pina-Martins and Paulo, 2008). Phylogenetic analyses of the sequence dataset were performed with maximum likelihood (ML) using PAUP*4b10 (Wilgenbusch and Swofford, 2003) and Bayesian inference (BI) using MrBayes 3.1.2 (Huelsenbeck and Ronquist, 2001). The best-fitted model of DNA evolution was obtained using ModelTest v.2 (Darriba *et al.* 2012) with the Akaike Information Criterion (AIC). The Akaike-supported model, base frequency, proportion of invariable sites, gamma distribution shape parameters and substitution rates in the AIC were then used in phylogenetic analyses. BI analysis under the TVM+G model for D3 expansion segment of 28S and the TVM+I+G model for 18S rDNA was initiated with a random starting tree and run with four Metropolis-coupled Markov chain Monte Carlo (MCMC) for 1×10^6^ generations in the combined dataset. The MCMC were sampled every 100 generations. Two runs were performed for each analysis. After discarding burn-in samples and evaluating convergence, the remaining samples were retained for further analyses. The topologies were used to generate a 50% majority rule consensus tree. Trees were visualized using TreeView (Page, 1996). In ML analysis the estimation of the support for each node was obtained by bootstrap analysis with 1000 heuristic search replicates using the previously obtained models. Posterior probabilities and bootstrap support are given on respective clades.

### Free-living occurrence observation

Three rats were infected with 500 iL3s of *S. venezuelensis* or *S. ratti*. Faeces samples from each rat were collected at 8, 12, 14, 16 and 24 days post-infection (d.p.i.) for *S. venezuelensis* and at 8 d.p.i. for *S. ratti*. Faeces (approx. 0·5 g) were cultured on a 2% (w/v) agar plate at various temperatures (19, 25, or 30 °C) for 3 days and free-living male and female nematodes were counted. Three agar plates for each day and each condition were used. A portion of the faecal samples (*c*. 1·5 g) was diluted in distilled water to determine the eggs/larvae per gram (epg/lpg) of faeces. All statistical analyses were performed using the software package R version 2.15.2 (http://www.r-project.org).

### Early embryogenesis

Parasitic females were obtained from rat intestine at 5–10 d.p.i. Fresh parasites were transferred to a pre-warmed (37 °C) agarose pad (4% (w/v) agarose) prepared on a microscope slide (Shaham, 2006) and covered with a silicon grease-rimmed cover slip and viewed under a Nomarski microscope (IX71, Olympus, Japan). All procedures involving the microscopic observations were performed at 37 °C.

### DAPI staining/microscopy

Parasitic females were fixed with ice-cold methanol and stained with DAPI as previously described (Nemetschke *et al.* 2010). To collect the eggs, parasitic females were allowed to lay eggs in PBS, the eggs were then squashed and stained as previously described with the exception of using methanol instead of acetic acid for fixation (Albertson *et al.* 1979). Microscopic observations were carried out using a confocal laser scanning microscope (LSM700, Zeiss).

## RESULTS

### Phylogenetic position of *S. venezuelensis*

**Fig. 2.**
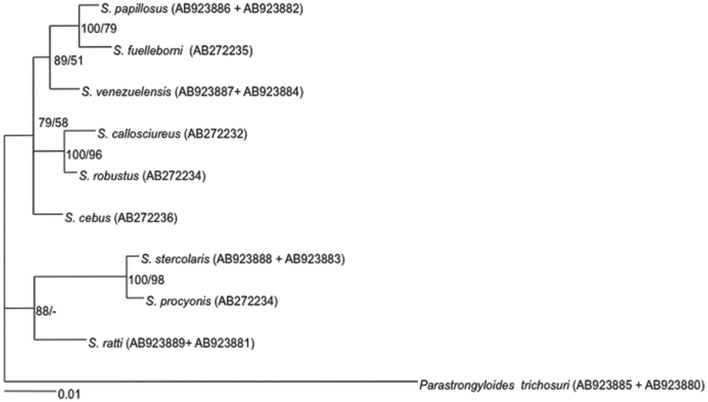
The molecular phylogenetic relationship between *Strongyloides* species inferred from concatenated nearly full-length 18S rRNA gene (1640 positions from edited alignment) and D3 expansion segments of 28S rRNA gene (289 positions from edited alignment). TVM+G and TVM+I+G were used for D3 expansion segment of 28S and 18S, respectively. Posterior probabilities (first number in the node label) more than 65% are given for appropriate clades; bootstrap values greater than 50% are given on appropriate clades in ML analysis (second number in the node label).

In order to determine the phylogenetic position of *S. venezuelensis* in the genus, we used nearly full-length 18S rRNA and D3 expansion segments of 28S rRNA genes. We sequenced these regions in *S. venezuelensis, S. papillosus, S. stercoralis, S. ratti* and *P. trichosuri* for phylogenetic analysis. 18S sequences from other *Strongyloides* species obtained from the public database were also included in the analysis. Our phylogenetic tree using *P. trichosuri* as an outgroup indicated that the *Strongyloides* species can be divided into two clades (Fig. 2): one clade comprising of *S. papillosus, S. fuelleborni, S. venezuelensis, Strongyloides callosciureus, Strongyloides robustus* and *Strongyloides cebus*, and the other including *S. stercoralis, Strongyloides procyonis* and *S. ratti*. Within the former clade, *S. venezuelensis* was further clustered into a subclade with *S. papillosus* and *S. fuelleborni*.

### Occurrences of free-living adults in *S. venezuelensis*

As the ratio of free-living females/males *vs* females developing homogonically into iL3s is influenced by several factors, including host immunity and other environmental conditions (temperature, pH etc.), in other *Strongyloides* species (Arizono, 1976; Moncol and Triantaphyllou, 1978; Nwaorgu, 1983; Viney, 1996; Harvey *et al.* 2000; Minato *et al.* 2008; Sakamoto and Uga, 2013), we used two parameters (days post infection and faeces incubation temperature) to examine the occurrence of free-living nematodes (FLNs) in *S. venezuelensis*.

In a total of 162 attempts, comprising of six d.p.i.s (8, 12, 14, 16, 20 and 24) and three temperature conditions (19, 25 and 30 °C), each with three rats and triplicate, we observed FLNs of *S. venezuelensis* in only 19 cases. As this frequency is much lower than that reported in *S. ratti* (Harvey *et al.* 2000; Minato *et al.* 2008; Sakamoto and Uga, 2013), we validated our method using *S. ratti* in place of *S. venezuelensis*. In the *S. ratti* experiment we observed FLNs in all culture plates tested (Table S1) and the numbers of FLNs (8·32 to 17·4 FLNs per 1000 eggs) were similar to those reported previously (Minato *et al.* 2008). Therefore we confirmed that this low frequency (19 out of 162 cases) was not due to errors in handling of the samples. Of note is that in some culture plates (52 cases) we did not observe the homogonically developed nematodes (iL3s) although the faeces contained a sufficient number of eggs, which may suggest that the development of *S. venezuelensis* is more sensitive to environmental conditions than that of *S. ratti*. Furthermore, free-living males were never detected in *S. venezuelensis* (Table 1) while approximately half of FLNs in the *S. ratti* experiment were males (Table S1). Consequently, no hatching was observed in eggs derived from the free-living females (data not shown).

**Table 1.** Total number of free living nematodes observed in *S. venezuelensis* faeces samples

	19 °C	25 °C	30 °C
Free-living female	0 (0/54)	17 (7/54)	46 (12/54)
Free-living male	0 (0/54)	0 (0/54)	0 (0/54)

Fifty-four culture plates were used at each temperature.

Numbers in parentheses represent numbers of incidents of free-living observation (i.e. 17(7/54) indicates a total number of 17 free-living nematodes found in 7 out of 54 culture plates).

In the 19 plates with *S. venezuelensis* FLNs, 7 plates (17 FLNs in total) were from 25 °C culture and 12 plates (46 FLNs) were from 30 °C culture (Table 1). We did not observe any FLNs in 19 °C cultures (Table 1).

**Fig. 3.**
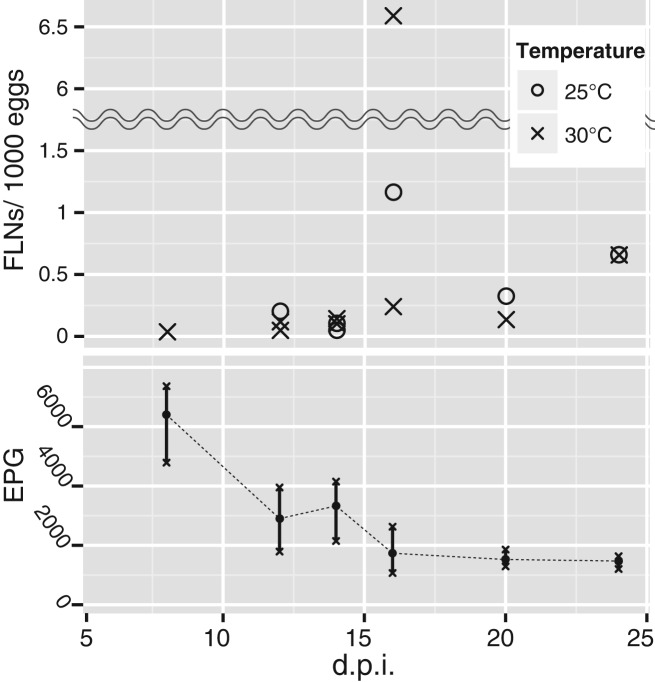
(Upper) Free-living occurrences in *S. venezuelensis*. Nineteen positive cultures (in which free-living nematodes were found) out of 162 were plotted by number of free-living nematodes (FLNs) per 1000 eggs and day post infection (d.p.i.). Faeces were cultured at 19, 25 or 30 °C. No free-living nematodes were observed in 19 °C cultures. (Bottom) EPG change by d.p.i. The black dot represents the mean and crosses represent the highest and the lowest in each d.p.i.: *n* = 3.

We observed *S. venezuelensis* FLNs in 1, 3, 4, 3, 2, and 2 cases at 8, 12, 14, 16, 20 and 24 d.p.i., respectively (Fig. 3) and the number of FLNs on each d.p.i. was 3, 6, 6, 43, 4, and 4, respectively. Although the number of eggs per gram of faeces (epg) decreased as the d.p.i. increased (Fig. 3), the number of instances that FLNs were observed did not change significantly (Generalized Linear Model with binomial error distribution: d*f* = 52, *P*>0·9). Then, we sought to investigate the relationship between epg and occurrence of FLNs. Figure S1 shows a plot of epg and number of FLNs per gram of faeces. The trend observed was that fewer epg showed more numbers of FLNs per egg (Fig. S1).

The highest number of FLNs was obtained with faeces collected at 16 d.p.i. and cultured at 30 °C (34 FLNs, 6·58 per 1000 eggs). However this was an outlier as the other two plates under the same conditions (same rat, d.p.i. and temperature) showed much lower numbers of FLNs (0·24 or 0 per 1000 eggs).

### Germ cells in the parasitic female

**Fig. 4.**
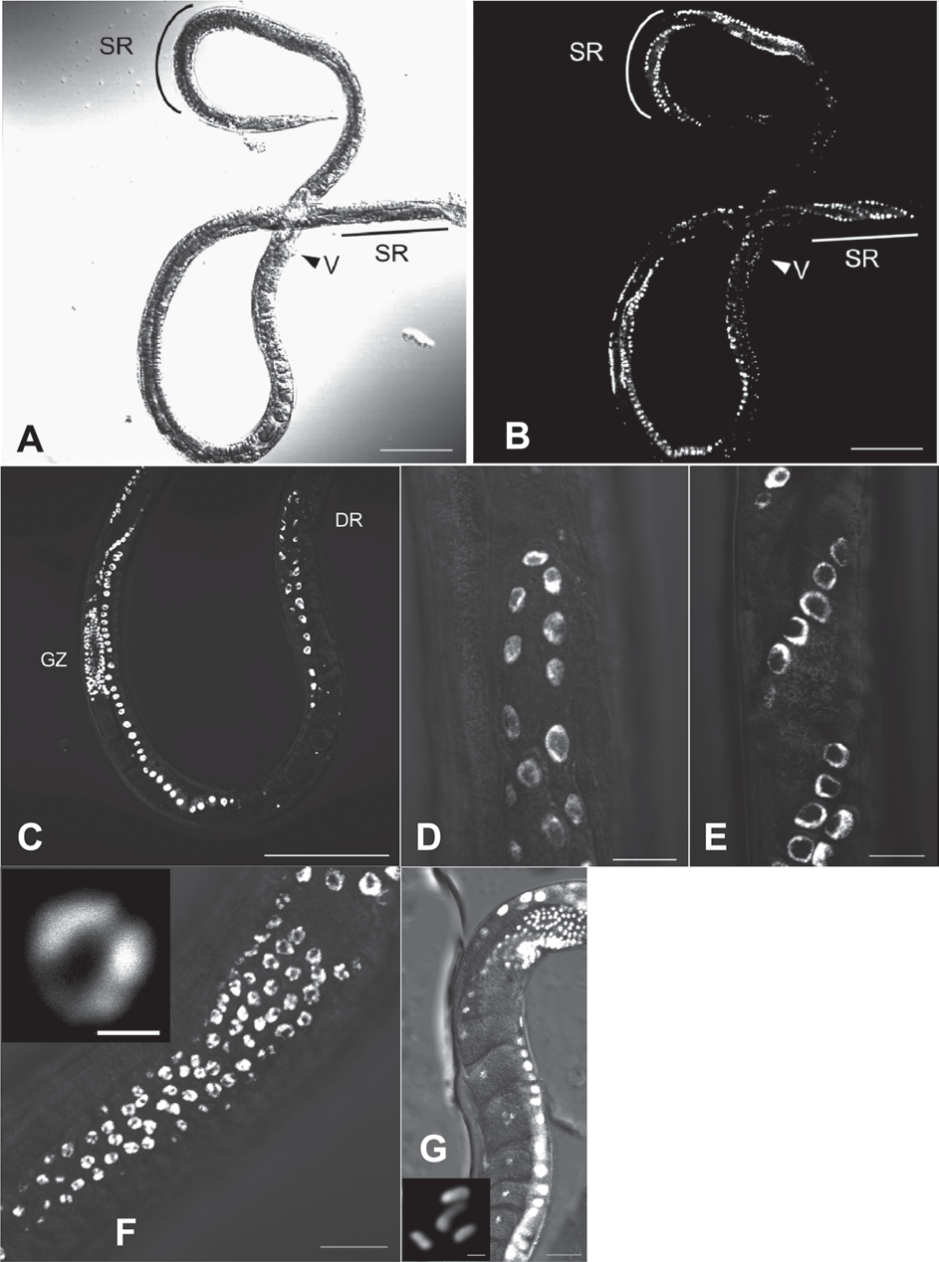
Germ cells in a parasitic female of *S. venezuelensis*. (A, B) Whole body of a parasitic female under bright field and DAPI fluorescence microscopy. V, vulva; SR, spiralled region; (C) Distal side of a gonad. DR: distal region GZ: germinal zone; (D) Oogonium in distal end of a gonad; (E) Oogonium in spiralled region of a gonad; (F) Condensed nucleus in the germinal zone; (G) Condensed chromosomes in the growth zone. (Scale bars: A, B, C = 100 *μ*m; D, E, F = 10 *μ*m; G = 20 *μ*m; boxes in F, G = 1 *μ*m).

*Strongyloides venezuelensis* parasitic females have two elongated (didelphic) gonads extending from the vulva to the head or the tail directions, reflexed around the beginning of the intestine or anus, continuing around the vulva (Little, 1966) (Fig. 4A, B). Egg development and embryogenesis progress as stages move along the tract from the germinal tissue to the vulva. Both gonads spiral about themselves around the intestine (Fig. 4A, B, E). As with many nematodes the distal part of the germ line was a syncytium; nuclei were located at the inner surface of the germ line (Fig. 4C–E). Chromosomes in all nuclei in the distal region dispersed peripherally, and nuclei were condensed in the ‘germinal zone’ (Fig. 4F). As nuclei moved away from the germinal zone they were packed one by one into a cell in the ‘growth zone’ and became oocytes (Fig. 4G). Germinal vesicles appeared and chromosomes became condensed during the oocyte movement towards the proximal region, and they seemed to be arrested at this stage until they passed through the oviduct (Fig. 4G). We detected four chromosomes and this state could be prometaphase of the ‘maturation division’ (see below). We did not identify any sperm or sperm nuclei in the gonad in either bright-field or DAPI-stained observations. From these results, we conclude that parasitic females of *S. venezuelensis* reproduce parthenogenetically.

### Early embryogenesis of eggs from the parasitic female

**Fig. 5.**
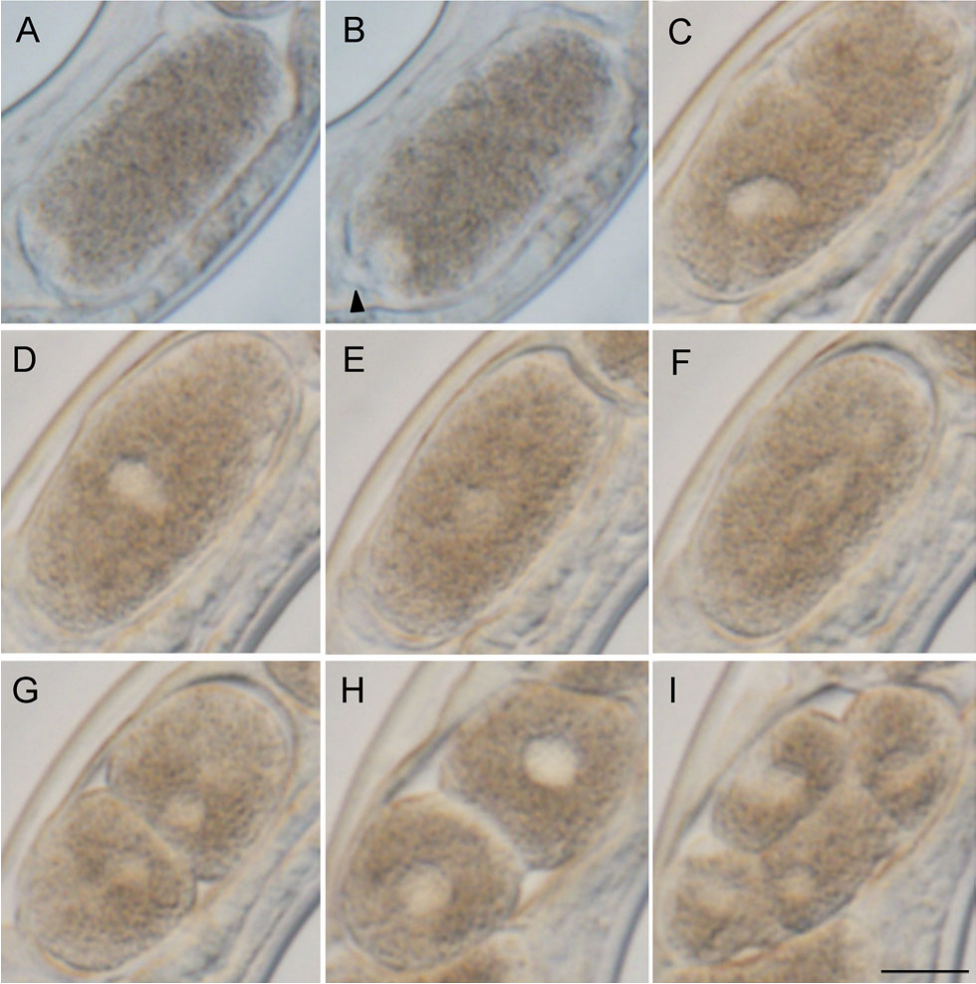
Early embryogenesis in a live *S. venezuelensis* parasitic female. The leading edge of the embryo is arranged to be upper right. (A) An embryo that has passed through the oviduct; (B) An embryo under maturation division; (C) Pseudocleavage with one pronucleus; (D) Nucleus moving to the centre of the cell; (E) Nucleus dispersed at the centre of the cell; (F, G, H) Cell division to form a two-cell embryo; (I) Four-cell embryo (Arrow head indicates a polar body, Scale bar = 10 *μ*m). Time frame of the development is shown in Table S2.

To see if the embryo starts development without fertilization, we observed oocyte maturation and early embryogenesis in the parasitic female sequentially with light microscopy. After the mature oocyte passed through the oviduct into the uterus, the eggshell was formed (Fig. 5A). The embryo shape was oval and the long axis of the embryo in the uterus was in parallel to the anterior-posterior axis of the mother. The germinal vesicle disappeared, and then only one pronucleus appeared at the lagging side-pole of the embryo (Fig. 5A, B). At this stage a protruded polar body-like structure was observed adjacent to the pronucleus (Fig. 5B). Pseudocleavage furrow and cytoplasmic streaming were observed (Fig. 5C), the pronucleus moved towards the middle of the cell (Fig. 5D) and its membrane broke down (Fig. 5E, F). Subsequently the embryo divided to form the two-cell stage (Fig. 5G, H) and the four-cell stage (Fig. 5I). All embryos (*n* = 7) observed in these experiments developed to first-stage larvae and hatched successfully (not shown), suggesting that the process observed was normal development.

### Chromosome structure and behaviour during early embryogenesis

**Fig. 6.**
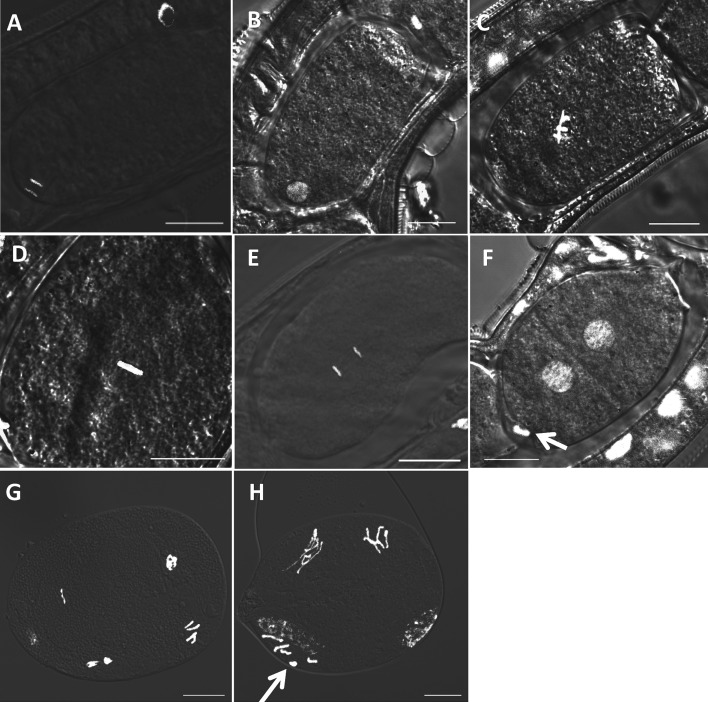
Chromosome behaviour after passing through oviduct into the uterus. The leading edge of the embryo is arranged to be upper right. (A) Anaphase of maturation division; (B) A pronucleus reconstructed after maturation division; (C) A pronucleus moves to the centre and chromosomes are formed; (D) Metaphase of the first embryonic cell division; (E) Anaphase of the first embryonic cell division; (F) Telophase of the first embryonic cell division; (G, H) Four cell embryos. Newly laid eggs were squashed and stained for the observation. (Arrows indicate polar bodies. All scale bars  = 10 *μ*m).

To clarify the mechanism by which ploidy is maintained, we observed chromosome structure and behaviour during early embryogenesis by DAPI staining. Developmental stages of the fixed and stained embryos were decided by comparison to the living embryos observed above. After passing through the oviduct into the uterus, the oocyte resumed mitotic cell division (Fig. 6A). This division always occurred asymmetrically at the lagging side-pole of the embryo (Fig. 6A) to produce one polar body. Polar bodies stained clearly as blue dots, but often disappeared as embryogenesis progressed. One pronucleus was reconstructed with vesicle (Fig. 6B), moved to the centre, and chromosomes were visible in the prophase stage (Fig. 6C). Chromatids fused and aligned along the metaphase plate (Fig. 6D) and migrated to each pole (Fig. 6E) subsequently forming two-cell stage embryos (Fig. 6F). The cleavage occurred asymmetrically with a bigger blastomere in the leading pole than the lagging one, which is contrary to the observations in *Caenorhabditis elegans* (Wallenfang and Seydoux, 2000) and other *Caenorhabditis* nematodes (Brauchle *et al.* 2009). Most eggs were laid as two-cell stage embryos. Then, we collected the laid eggs, squashed and stained with DAPI. Four chromosomes, two longer than the others, were observed in prophase cells in the four-cell stage embryos (Fig. 6G, H), suggesting that the karyotype of *S. venezuelensis* is 2*n* = 4. We confirmed in nine eggs, all of which had four chromosomes, and no specimens showed different compositions of chromosomes.

## DISCUSSION

*Strongyloides* spp. are unique among parasitic nematodes in having both parasitic and free-living stages in their life cycle. Since the parasitic females produce eggs by parthenogenesis, the free-living stage is the only occasion for them to exchange genetic materials with other individuals. Eggs that are parthenogenetically produced by the parasitic females can develop into three morphs: free-living males, free-living females or iL3. In *S. ratti* the frequency of free-living development is as high as 60% (Minato *et al.* 2008), which suggests frequent exchanges of genetic materials take place in this species. In this study we observed a much lower frequency of free-living development in *S. venezuelensis* than *S. ratti*. Furthermore, we did not find any free-living males. This suggests that *S. venezuelensis* rarely, or possibly never, exchanges genetic materials with other individuals in its life cycle. It is still possible, however, that there are unknown triggers to stimulate the nematode to have more free-living males and enable them to perform genetic exchanges. This could be an artificial loss of ability due to the stress of prolonged maintenance in laboratory, though absence of free-living males was reported previously in a recent field isolate of *S. venezuelensis* (Hasegawa *et al.* 1988). It would be of value to investigate free-living occurrence rates in other strains or wild isolates to confirm if this is a general characteristic of the species or the observation in this study is an exceptional case.

We noted that the number of free-living females increased with days after infection, although it was not clear which parameter was more influential, days post infection (i.e. ageing of worms and effect of host immunity) or dispersed density of eggs in the faeces. We also observed a higher number of free-living females in 30 and 25 °C cultures than at 19 °C. A similar trend for temperature conditions was also observed with other *Strongyloides* species (Arizono, 1976; Nwaorgu, 1983; Minato *et al.* 2008; Sakamoto and Uga, 2013).

We observed four chromosomes in *S. venezuelensis* somatic cells, suggesting *S. venezuelensis* has a karyotype of 2*n* = 4. It is known that *S. ratti* has three pairs of chromosomes (2*n* = 6) and one of them is a sex chromosome. On the other hand, *S. papillosus* females have two pairs of chromosomes (2*n* = 4). The lengths of the two are significantly different and the longer one is suggested to be the result of a fusion of a sex chromosome with one of the autosomes of 2*n* = 6 (Triantaphyllou and Moncol, 1977). The number of chromosomes in *S. venezuelensis* could also result from such a fusion. However, the lengths of the two pairs of the four *S. venezuelensis* chromosomes did not differ from each other to the extent seen in *S. papillosus* (Nemetschke *et al*. 2010). It would be very interesting to correlate the evolutionary history of reproduction in *Strongyloides* species with their chromosome structures.

Nemetschke *et al.* showed that *S. papillosus* employs chromatin diminution to exclude chromosome regions corresponding to the *S. ratti* sex chromosome in males (Albertson *et al.* 1979; Nemetschke *et al.* 2010). Although *S. venezuelensis* has the same number of chromosomes as *S. papillosus* and chromosome diminution could generate males in this species, we did not observe such events or any different chromosome composition in this study. This correlates with the absence of males in *S. venezuelensis*, although the basis for the lack of chromatin diminution is unclear: it could be that we were unable to generate *S. venezuelensis* males via this procedure or that males were not observed because *S. venezuelensis* is not capable of chromatin diminution.

Because embryogenesis occurred without fertilization and the chromosome number in mature oocytes, which are located proximal to the oviduct, was the same as that of the somatic cells it is likely the *S. venezuelensis* reproduces via mitotic parthenogenesis. Even without sperm stimulation, an embryo of *S. venezuelensis* had a polarity and produced the polar body on the lagging pole of the embryo. This orientation is opposite from *C. elegans* (Wallenfang and Seydoux, 2000). Parasitic stages of *Strongyloides* species including *S. ratti* or *S. papillosus* have been reported to have only female sex and reproduce by mitotic parthenogenesis (Zaffagnini, 1973; Triantaphyllou and Moncol, 1977; Viney, 1994). Our observation is consistent with these reports. In order to confirm this, molecular studies would be useful by checking the progenies have the same genotype (clones) or have genetic variations due to cross-overs of sister chromosomes.

Our phylogenetic analysis using genes for 18S and D3 of 28S ribosomal RNA showed two well-supported clusters in *Strongyloides* species, namely a group including *S. ratti* and *S. stercoralis* and another including *S. papillosus* and *S. venezuelensis* (Fig. 2). This is consistent with the gonad morphologies of these species, wherein members of the *S. papillosus* group have spiral morphology, while those of the *S. ratti* group have straight morphology (Bartlett, 1995; Little, 1966; Sato *et al.* 2007). The number of chromosomes observed for *Strongyloides* species was also consistent with the phylogeny; karyotypes known thus far are 2n = 6 for *S. ratti* and *S. stercoralis* and 2n = 4 for *S. papillosus* and *S. venezuelensis. Strongyloides ratti* and *S. venezuelensis* are both parasites of rodents. However they are clearly different in terms of rRNA phylogeny, chromosome number and gonad morphology. Therefore, it is likely that they acquired their ability to parasitize rodents independently from each other.

## Supplementary material

For supplementary material accompanying this paper visit http://dx.doi.org/10.1017/S0031182014001036. click here to view supplementary material

## DATA DEPOSITION

Sequence data has been deposited in GenBank/EMBL/DDBJ under accession numbers AB923880–923889.

## References

[ref1] Albertson D. G., Nwaorgu O. C. and Sulston J. E. (1979). Chromatin diminution and a chromosomal mechanism of sexual differentiation in *Strongyloides papillosus*. Chromosoma 75, 75–87. 533664 10.1007/BF00330626

[ref2] Arizono N. (1976). Studies on the free-living generations of *Strongyloides planiceps*, 1943 II. Effect of temperature on the developmental types. Japanese Journal of Parasitology 25, 328–335.

[ref3] Bartlett C. M. (1995). Morphology, homogonic development, and lack of a free-living generation in *Strongyloides robustus* (Nematoda, Rhabditoidea), a parasite of North American sciurids. Folia Parasitologica 42, 102–114. 8774766

[ref4] Brauchle M., Kiontke K., Macmenamin P., Fitch D. H. and Piano F. (2009). Evolution of early embryogenesis in rhabditid nematodes. Developmental Biology 335, 253–262. 19643102 10.1016/j.ydbio.2009.07.033 PMC2763944

[ref5] Brumpt E. (1934). Précis de Parasitologie, 6th Edn Masson et Cie, Paris.

[ref6] Chitwood B. and Graham G. (1940). Absence of vitelline membranes on developing eggs in parasitic females of *Strongyloides ratti*. Journal of Parasitology 26, 183–190.

[ref7] Darriba D., Taboada G. L., Doallo R. and Posada D. (2012). jModelTest 2: more models, new heuristics and parallel computing. Nature Methods 9, 772. 22847109 10.1038/nmeth.2109 PMC4594756

[ref8] Dorris M., Viney M. E. and Blaxter M. L. (2002). Molecular phylogenetic analysis of the genus *Strongyloides* and related nematodes. International Journal for Parasitology 32, 1507–1517. 12392916 10.1016/s0020-7519(02)00156-x

[ref9] Eberhardt A. G., Mayer W. E. and Streit A. (2007). The free-living generation of the nematode *Strongyloides papillosus* undergoes sexual reproduction. International Journal for Parasitology 37, 989–1000. 17324432 10.1016/j.ijpara.2007.01.010

[ref10] El-Malky M. A., Maruyama H., Al-Harthi S. A., El-Beshbishi S. N. and Ohta N. (2013). The role of B-cells in immunity against adult *Strongyloides venezuelensis*. Parasites and Vectors 6, 148. 23705584 10.1186/1756-3305-6-148 PMC3669613

[ref11] Harvey S. C., Gemmill A. W., Read A. F. and Viney M. E. (2000). The control of morph development in the parasitic nematode *Strongyloides ratti*. Proceedings of the Royal Society B: Biological Sciences 267, 2057–2063. 10.1098/rspb.2000.1249 PMC1690777 11416909

[ref12] Hasegawa H., Orido Y., Sato Y. and Otsuru M. (1988). *Strongyloides venezuelensis* Brumpt, 1934 (Nematoda: Strongyloididae) collected from *Rattus norvegicus* in Naha, Okinawa, Japan. Japanese Journal of Parasitology 37, 429–434.

[ref13] Holterman M., Van Der Wurff A., Van Den Elsen S., Van Megen H., Bongers T., Holovachov O., Bakker J. and Helder J. (2006). Phylum-wide analysis of SSU rDNA reveals deep phylogenetic relationships among nematodes and accelerated evolution toward crown clades. Molecular Biology and Evolution 23, 1792–1800. 16790472 10.1093/molbev/msl044

[ref14] Huelsenbeck J. P. and Ronquist F. (2001). MrBayes: Bayesian inference of phylogenetic trees. Bioinformatics 17, 754–755. 11524383 10.1093/bioinformatics/17.8.754

[ref15] Katoh K., Misawa K., Kuma K. and Miyata T. (2002). MAFFT: a novel method for rapid multiple sequence alignment based on fast Fourier transform. Nucleic Acids Research 30, 3059–3066. 12136088 10.1093/nar/gkf436 PMC135756

[ref16] Little M. D. (1966). Comparative morphology of six species of *Strongyloides* (Nematoda) and redefinition of the genus. Journal of Parasitology 52, 69–84. 5929983

[ref17] Matsumoto M., Sasaki Y., Yasuda K., Takai T., Muramatsu M., Yoshimoto T. and Nakanishi K. (2013). IgG and IgE collaboratively accelerate expulsion of *Strongyloides venezuelensis* in a primary infection. Infection and Immunity 81, 2518–2527. 23630966 10.1128/IAI.00285-13 PMC3697603

[ref18] Minato K., Kimura E., Shintoku Y. and Uga S. (2008). Effect of temperature on the development of free-living stages of *Strongyloides ratti*. Parasitology Research 102, 315–319. 18026994 10.1007/s00436-007-0773-7

[ref19] Moncol D. J. and Triantaphyllou A. C. (1978). *Stronglyoides ransomi*: factors influencing the *in vitro* development of the free-living generation. Journal of Parasitology 64, 220–225. 25317

[ref20] Nemetschke L., Eberhardt A. G., Hertzberg H. and Streit A. (2010). Genetics, chromatin diminution, and sex chromosome evolution in the parasitic nematode genus *Strongyloides*. Current Biology 20, 1687–1696. 20832309 10.1016/j.cub.2010.08.014

[ref21] Nunn G. B., Theisen B. F., Christensen B. and Arctander P. (1996). Simplicity-correlated size growth of the nuclear 28S ribosomal RNA D3 expansion segment in the crustacean order Isopoda. Journal of Molecular Evolution 42, 211–223. 8919873 10.1007/BF02198847

[ref22] Nwaorgu O. C. (1983). The development of the free-living stages of *Strongyloides papillosus*. I. Effect of temperature on the development of the heterogonic and homogonic nematodes in faecal culture. Veterinary Parasitology 13, 213–223. 6686378 10.1016/0304-4017(83)90058-4

[ref23] Page R. D. (1996). TreeView: an application to display phylogenetic trees on personal computers. Computer Applications in the Biosciences 12, 357–358. 8902363 10.1093/bioinformatics/12.4.357

[ref24] Pina-Martins F. and Paulo O. S. (2008). Concatenator: sequence data matrices handling made easy. Molecular Ecology Resources 8, 1254–1255. 21586013 10.1111/j.1755-0998.2008.02164.x

[ref25] Sakamoto M. and Uga S. (2013). Development of free-living stages of *Strongyloides ratti* under different temperature conditions. Parasitology Research 112, 4009–4013. 24043614 10.1007/s00436-013-3591-0

[ref26] Sato H., Torii H., Une Y. and Ooi H. K. (2007). A new rhabditoid nematode species in Asian sciurids, distinct from *Strongyloides robustus* in North American sciurids. Journal of Parasitology 93, 1476–1486. 18314696 10.1645/GE-1106.1

[ref27] Sato Y. and Toma H. (1990). Effects of spleen cells and serum on transfer of immunity to *Strongyloides venezuelensis* infection in hypothymic (nude) mice. International Journal for Parasitology 20, 63–67. 2312228 10.1016/0020-7519(90)90174-l

[ref28] Shaham S. (2006). Methods in Cell Biology. *WormBook*, The *C. elegans* Research Community. doi: 10.1895/wormbook.1.49.1.

[ref29] Streit A. (2008). Reproduction in Strongyloides (Nematoda): a life between sex and parthenogenesis. Parasitology 135, 285–294. 18076772 10.1017/S003118200700399X

[ref30] Takamure A. (1995). Migration route of *Strongyloides venezuelensis* in rodents. International Journal for Parasitology 25, 907–911. 8550290 10.1016/0020-7519(95)00014-s

[ref31] Triantaphyllou A. C. and Moncol D. J. (1977). Cytology, reproduction, and sex determination of *Strongyloides ransomi* and *S. papillosus*. Journal of Parasitology 63, 961–973. 592051

[ref32] Viney M. E. (1994). A genetic analysis of reproduction in *Strongyloides ratti*. Parasitology 109, 511–515. 7800419 10.1017/s0031182000080768

[ref33] Viney M. E. (1996). Developmental switching in the parasitic nematode *Strongyloides ratti*. Proceedings of the Royal Society B: Biological Sciences, 263, 201–208. 10.1098/rspb.1996.0032 8728983

[ref34] Viney M. E. and Lok J. B. (2007). *Strongyloides* spp. WormBook 1–15. doi: 10.1895/wormbook.1.141.1. 18050491 PMC3091011

[ref35] Wallenfang M. R. and Seydoux G. (2000). Polarization of the anterior-posterior axis of *C. elegans* is a microtubule-directed process. Nature 408, 89–92. 11081513 10.1038/35040562

[ref36] Wertheim G. and Lengy J. (1964). The seasonal occurrence of *Strongyloides ratti* sandground, 1925 and of S. Venezuelensis Brumpt, 1934 in a population of *Rattus norvegicus*. Journal of Helminthology 38, 393–398. 14250824 10.1017/s0022149x00033940

[ref37] Wilgenbusch J. C. and Swofford D. (2003). Inferring evolutionary trees with PAUP*. *Current Protocols in Bioinformatics*, Chapter 6, Unit 6.4. doi: 10.1002/0471250953.bi0604s00. 18428704

[ref38] Wilkes C. P., Bleay C., Paterson S. and Viney M. E. (2007). The immune response during a *Strongyloides ratti* infection of rats. Parasite Immunology 29, 339–346. 17576363 10.1111/j.1365-3024.2007.00945.x PMC2042580

[ref39] Yamada M., Matsuda S., Nakazawa M. and Arizono N. (1991). Species-specific differences in heterogonic development of serially transferred free-living generations of *Strongyloides planiceps* and *Strongyloides stercoralis*. Journal of Parasitology 77, 592–594. 1865267

[ref40] Zaffagnini F. (1973). Parthenogenesis in the parasitic and free-living forms of *Strongyloides papillosus* (Nematoda, Rhabdiasoidea). Chromosoma 40, 443–450. 4693091 10.1007/BF00399433

