# Supplementary material for: Karyotype and reproduction mode of the rodent parasite Strongyloides venezuelensis
Source: Parasitology. 2014 Aug 4;141(13):1736–45. doi: 10.1017/S0031182014001036 (PMC4183129; doi:10.1017/S0031182014001036)
Supplement: Supplementary file 1 [file S0031182014001036sup.zip › S0031182014001036sup/S0031182014001036sup001.pdf]

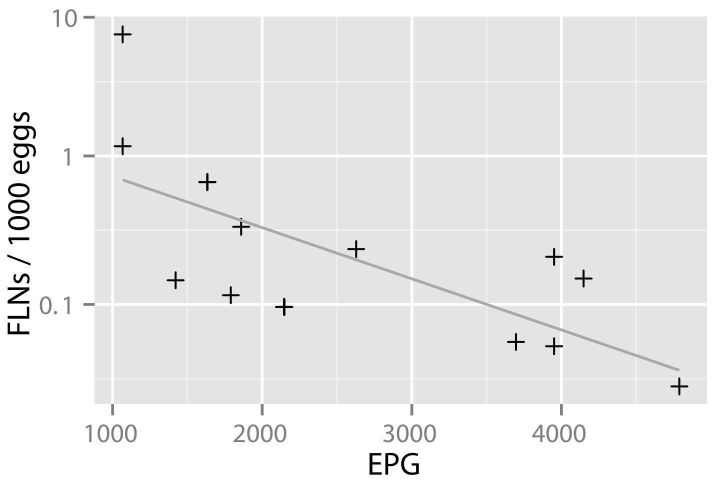

Fig. S1. The correlation between number of free-living nematodes and EPG. Nineteen positive cultures were plotted by number of free-living nematodes per 1000 eggs and EPG. The gray line indicates the best fit linear regression (y=-3.45e-04x +2.07, r^2=0.50, p<0.01, df=13).
