# Supplementary material for: Karyotype and reproduction mode of the rodent parasite Strongyloides venezuelensis
Source: Parasitology. 2014 Aug 4;141(13):1736–45. doi: 10.1017/S0031182014001036 (PMC4183129; doi:10.1017/S0031182014001036)
Supplement: Supplementary file 1 [file S0031182014001036sup.zip › S0031182014001036sup/S0031182014001036sup002.pdf]

Table S1. Number of free living nematodes observed in *S. ratti* feces of 8 days pos

	Rat1	Rat2	Rat3
Free living female	1875 (3/3)	3110 (3/3)	2550 (3/3)
(per 1000 eggs/larva)	9.83	17.4	8.32
Free living male	1800 (3/3)	3740 (3/3)	2285 (3/3)
(per 1000 eggs/larva)	9.44	20.0	8.18

Free-living nematodes were observed in all culture plates tested.
Numbers in parenthes represent numbers of incidents of free-living observation.

TableS2. Time required between each key stages of *S. venezuelensis* embryo at 37ºC

Developmental Stage	Corresponding figures	Time (min)	
pseudocleavage - nucleus migration to center	Fig 5 C -D	3.18±1.36	n=4
nucleus migration to center - nucleus disappearance	Fig 5 D - F	2.38±1.36	n=6
nucleus disappearance - two cells	Fig 5 F - G	2.64±1.12	n=7
two cells - nucleus re-appearance	Fig 5 G - H	2.78±1.07	n=8
nucleus re-appearance - four cells	Fig 5 H - I	11.1±2.11	n=4

± : standard deviation
